# Psychological Impact on Firefighters After the 2022 Amok Attack in Berlin at Tauentzienstraße

**DOI:** 10.3390/healthcare13030263

**Published:** 2025-01-29

**Authors:** Francesco Pahnke, Nils Hüttermann, Jan Philipp Krüger, Ulrich Wesemann

**Affiliations:** 1 Department of Psychiatry, Psychotherapy and Psychotraumatology, Bundeswehr Hospital Berlin, Scharnhorststr. 13, 10115 Berlin, Germany; 2 Department of Microbiology, Bundeswehr Hospital Berlin, 10115 Berlin, Germany

**Keywords:** emergency responders, intentional vehicular assault, panic disorder, mental health, firefighters

## Abstract

Objective: Exposure of emergency service personnel to disasters can lead to significant mental health challenges. The psychological impact of intentionally caused disasters, such as terrorist attacks, tends to be more severe than that of natural disasters. While much research has focused on terrorist attacks, little is known about the effects of intentional vehicular assaults (IVAs). This study examines the impact of an IVA on the mental health of firefighters. We hypothesized that firefighters deployed to the scene (deployed group (DG)) would experience more mental health problems compared to those not on duty (comparison group (CG)). Methods: The study included *n* = 115 firefighters, with 60 in the DG and 55 in the CG from the same units. Validated psychometric tools were used to assess anxiety, panic attacks (PHQ-D), and post-traumatic stress symptoms (PCL-5). Participation was voluntary, and informed consent was obtained. The study received approval from the Charité Berlin Ethics Committee (number: EA4/085/18). Results: A significantly higher prevalence of panic attacks was found in the DG (12.5%) compared to the CG (1.8%), with an odds ratio of 8.0 (95% CI: 1.0–67.3). Correlation analysis revealed a significant positive relationship between non-occupational tasks and hostility (*r* = 0.312, *p* = 0.015, *n* = 60), while parenthood had no significant effect on panic attacks or generalized anxiety. Conclusion: These results highlight the severe mental health impact of intentional disasters like IVAs on firefighters, emphasizing the need for targeted psychological support and interventions. Future research should focus on tailored interventions to address the high prevalence of panic attacks among this population.

## 1. Introduction

Work in the field of emergency service personnel (ESP) is associated with a higher risk of physical and mental health issues compared to other occupational groups [1,2,3]. Along with common job-related stressors, such as shift work and the rapid transition between inactive and highly intense work periods, which are among the most prevalent stressors in ESP [4], emergency personnel are also more likely to encounter critical incidents, including workplace violence [2,4,5], particularly firefighters [6,7]. A 2019 survey found that 85% of emergency service personnel (ESP) had encountered a critical incident in the previous two months, with half witnessing one or more deaths during that time [8]. Such critical incidents, including witnessing violent deaths, performing resuscitations, or treating severely injured individuals, can be deeply distressing [9,10,11]. Incidents involving children, as well as accidents and suicides, are among the most distressing events [12]. Moreover, the mental health impact of intentional events, like attacks, tends to be more severe than that of accidents or natural disasters [13].

Research on the prevalence of post-traumatic stress disorder (PTSD) in ESP following incidents of human-made mass violence reveals significant variations. A meta-analysis reported PTSD prevalence rates among ESP responding to intentionally caused disasters ranging from 1.3% to 22.0%, illustrating significant heterogeneity in the field [1]. This variability appears not only across different types of ESP [1] but also among the victims involved in critical incidents [14]. National contexts also play a role, as ESP in different countries face unique challenges [6]. Systematic reviews further highlight this diversity, with PTSD prevalence among ESP ranging from 1.3% to 16.5% and anxiety disorders from 0.7% to 14% [15]. This wide range could be attributed to several factors, including the lack of specific training for critical events such as terrorist attacks, pandemics [16,17], and intentional vehicular assaults (IVAs) [18]. In Berlin, the Fire Department has implemented support programs, such as the Emergency Aftercare Team (ENT) [19], and training initiatives like the Charly BOS program [20]. But factors such as stressful life events occurring in the year prior to an incident [18], the severity of a critical incident (as seen with the very high prevalence of PTSD among responders following the 9/11 WTC attacks), and the distinctions between different professional groups and the specific tasks of emergency service personnel may also contribute to the observed variation in prevalence rates [15].

In previous studies, hostility has primarily been observed among military personnel or police officers in hostile environments. Given that hostility is frequently linked to mental health issues such as PTSD and depression, it would be valuable to explore whether firefighters, who are required to perform non-occupational tasks during critical incidents that are outside of their typical duties, such as taking on police responsibilities, may potentially develop hostility too [21,22,23].

Previous research has mainly focused on PTSD as the dominant psychological impact on firefighters following traumatic events (1, 2). However, panic attacks and generalized anxiety disorder (GAD), despite their established association with PTSD [24,25,26], have received comparatively little attention in studies involving ESP [27]. In 2018, researchers emphasized the need to investigate other mental health outcomes, suggesting that some may have been overlooked [28].

Our study aims to assess secondary trauma experienced by firefighters deployed to an intentional vehicular assault (IVA) in Berlin on 8 June 2022 [29]. During this incident, a driver deliberately targeted pedestrians, resulting in multiple casualties and complex response challenges. Firefighters treated 50 individuals on site, including students from a school group. One teacher lost their life [19].

The firefighters faced numerous challenges: the dynamic nature of the situation, the spatial spread of casualties typical of such incidents, and the need for rapid mass-casualty triage [30,31,32]. Additionally, IVAs often involve younger victims and more severe injuries than typical traffic accidents [29], compounded by the threat to responders themselves, such as a possible “second hit” from explosives in the vehicle [30], which could generate a hostile environment [33] and contribute to psychological burden [9,31,34].

The aim of the study was to compare the psychological burden experienced by firefighters who were deployed to the scene with those who were not. Specifically, we hypothesized that the deployed group would show higher levels of psychological distress compared to a non-deployed comparison group (H1). Based on previous findings suggesting that emergency service personnel (ESP) may experience a heightened emotional connection in such situations [9], we hypothesized that being a parent would be associated with increased psychological distress in these scenarios (H2).

Null hypotheses:

**Null** **Hypotheses** **(H0.1).** *There is no difference in psychological distress between deployed and non-deployed firefighters.*

**Null** **Hypotheses** **(H0.2).** *Parenthood does not influence psychological distress in first responders during emergencies involving children.*

## 2. Materials and Methods

### 2.1. Survey Strategy

The study used a written survey as the primary method of data collection and was conducted 6–8 months after the IVA due to ethical considerations, allowing participants time to process immediate stress and minimize the risk of retraumatization. This study focused on the first measurement point, with follow-up assessments planned at 12 and 24 months after the critical incident to evaluate long-term impact. The surveys were administered to the firefighters who were deployed during the IVA in Berlin in 2022 (deployed group (DG), *n* = 60) as well as a comparison group (CG) of firefighters from the same departments who were not deployed during the attack (*n* = 55).

### 2.2. Participants

The study included a total of 115 emergency service personnel (ESP), comprising 8 females (7.0%). Of these, 100 were from the Berlin Fire Department, including paramedics who also serve as firefighters for the department. At the time of the incident, 124 firefighters were present on-site (3). We successfully engaged 39.5% of the firefighters deployed during the IVA to participate in our survey, and 49% of the 100 firefighters we surveyed were in the DG.

### 2.3. Ethical Approval

The study was approved by the Institutional Review Board (Ethics Committee) of Charité Berlin (protocol code: EA4/085/18), and written informed consent was given by all subjects involved in the study.

### 2.4. Procedure

After receiving approval for our survey from the leadership of the Berlin Fire Department, we contacted all fire stations involved in the incident through their station leaders or unit commanders to arrange survey appointments. We made efforts to track down all firefighters to give them the opportunity to participate in our study, including those who were on vacation, had been transferred, or were absent for any other reason on the days of the survey. In addition to gathering the deployed firefighters (DG), we coordinated with the station leaders to identify other firefighters who were on duty at the station during the time of the survey but had not been deployed to the critical incident. These individuals formed the CG.

We made significant efforts to minimize any barriers to participation. With the consent of the Incident Aftercare Team (ENT) and the Berlin Fire Department leadership, all participants were temporarily relieved of duty to ensure they could be fully briefed and complete the survey without interruptions. They also had the opportunity to use various rooms of the fire station to complete the questionnaires in peace and without time pressure.

All firefighters were informed about the voluntary nature of their participation, the pseudonymization of the study, and the potential risk of short-term stress associated with completing the survey. Additionally, they were offered the opportunity to contact our Department of Psychotraumatology at the Bundeswehr Hospital Berlin at any time if they experienced any negative emotions or felt distressed after completing the questionnaire.

### 2.5. Measurement Instruments

All questionnaires used are self-report instruments and are available in a validated German-language version. The initial assessment included the Basic Questionnaire, which captured socio-demographic information and details about the respondents’ deployment activities.

The Posttraumatic Stress Disorder Checklist (PCL-5) [35] was employed to evaluate post-traumatic stress symptoms (PTSSs) and provisional PTSD diagnoses according to DSM-5 and ICD-11 criteria, with all questions referring to the past month. It is a 20-item questionnaire that uses a 5-point scale from 0 (“Not at all”) to 4 (“Extremely”). It is structured into subscales A–E. The total scores range from 0 to 80.

The Patient Health Questionnaire (PHQ-D) [28] was used to evaluate commonly occurring mental health disorders [36]. This included the assessment of generalized anxiety disorder, measured through 7 items (5a–5g) on a 4-point scale ranging from 0 (“Not at all”) to 3 (“Nearly every day”). Total scores range from 0 to 21.

Additionally, panic disorder was assessed using 15 items. A diagnosis can be confirmed if “Yes” is selected for all four questions in 3a–d and at least four of the questions in 4a–k also receive a “Yes” response [37].

The Brief Symptom Inventory (BSI) was used to assess hostility in firefighters over the past seven days. The general inventory comprises nine scales and a total of 53 items.

Scale 6 specifically focuses on aggression and hostility, including items 6, 13, 40, 41, and 46. This scale covers a range of emotions, from irritability and emotional instability to intense aggression with hostile traits. Responses are rated on a 5-point scale, ranging from 0 (“Not at all”) to 4 (“Extremely”) [38].

Statistical Analysis: Sociodemographic data were compared between the DG and CG using χ^2^ tests or *t*-tests for independent samples. Differences in the prevalence of mental disorders between the DG and the CG were assessed using χ^2^ tests, and odds ratios (ORs) were calculated. The same test was used to examine differences in major life events and differences in the prevalence of panic attacks and generalized anxiety in members of the DG with and without children. To examine the relationship between hostility and engagement in non-occupational tasks, we performed a correlation analysis. All statistical analyses were conducted using SPSS Statistics for Windows, Version 24.0 (IBM Corp., released 2016; IBM SPSS Statistics for Windows, Version 24.0. Armonk, NY, USA: IBM Corp.).

## 3. Results

Comparing both groups (DG and CG), there were no differences in sociodemographic data, as shown in Table 1. The age of the participants in this study ranged from 20 to 60, with a median age of 35.5 years and a mean age of 38 years.

### 3.1. Major Life Events in the Past 5 Years

We tested for differences in major life events in the past 5 years between the DG and CG to ensure that any potential impact of prior experiences on the study outcomes was evenly distributed across both groups. The chi-square test revealed no significant difference in the occurrence of these events between the two groups (χ^2^ = 0.07, *p* = 0.823).

### 3.2. Prevalence of Panic Attacks

To detect differences in the occurrence of panic attacks between the two groups, a chi-square test was conducted. The results χ^2^ (1, *n* = 113) = 4.96, *p* = 0.026, revealed a significantly higher proportion of individuals reporting panic attacks in the DG. The point prevalence of panic attacks was 12.5%, in contrast to 1.8% in the CG. The odds ratio (OR) of 8.0 (95% CI: 1.0–67.3) showed that the likelihood of experiencing panic attacks in the DG was eight times higher than in the CG.

### 3.3. Assessment of Pre-Existing Panic Attacks

We further investigated the occurrence of panic attacks to better understand the psychological impact of the incident and determine whether these attacks were pre-existing or triggered by the critical event. This was carried out using question 3b of the PHQ-D, which asks if panic attacks occurred before. None of the participants reported experiencing panic attacks prior to the event.

### 3.4. Prevalence of Generalized Anxiety

The chi-square test χ^2^ (1, *n* = 113) = 1.83, *p* = 0.176, indicated that no statistically significant differences emerged between the DG and the CG in relation to generalized anxiety. The point prevalence of generalized anxiety was 7.1% in the DG and 1.8% in the CG. The OR was 4.2 (95% CI: 0.4–38.4).

### 3.5. PTSD and Hostility

According to our findings, there were no reported cases of PTSD in either group (0%). We conducted a correlation analysis to examine the relationship between hostility and the assumption of non-occupational tasks. The results of the correlation analysis revealed a significant positive correlation (*r* = 0.312, *p* = 0.015, *n* = 60).

### 3.6. PCL-5 Subscores

To further investigate group differences in PTSD symptoms, we calculated independent *t*-tests for the PCL-5 subscores. As shown in Table 2, no significant differences were found between the study groups (DG and CG) in the individual subscores of Intrusions, Avoidance, and Negative Cognitions. However, the Arousal subscore (*p* = 0.041) and the Total PCL-5 score (*p* = 0.043) showed trends toward higher scores in the comparison group (CG). Despite these trends, the findings did not reach statistical significance after applying Bonferroni adjustment (significance level: *p* = 0.01).

### 3.7. Impact of Parenthood

To assess the impact of parenthood, a comparison was conducted between firefighters with and those without children. In the DG, 65% of participants had children, while in the CG, 58% had children. A chi-square test was performed to examine the prevalence of panic attacks in the two groups. The analysis showed no statistically significant difference in the prevalence of panic attacks between the firefighters with (13.5%) and without (10.2%) children (χ^2^ = 0.1, *p* = 0.749). Similarly, a chi-square test was conducted to assess the prevalence of GAD in the two groups. The analysis revealed no significant differences in the prevalence of GAD between the firefighters with (34.6%) and without (0%) children (χ^2^ = 2.21, *p* = 0.137).

## 4. Discussion

The results of our study indicate that the firefighters who responded to the IVA scenario (DG) in the line of duty exhibited a significantly higher rate of panic attacks than the CG (12.5% versus 1.8%). To account for prior trauma, we tested for major life events in the past five years and found no significant differences between the groups (χ^2^ = 0.07, *p* = 0.823). Additionally, none of the participants reported experiencing panic attacks before. These results suggest that the higher rate of panic attacks in the DG group is likely associated with the critical incident itself, rather than prior traumatic experiences or pre-existing panic attacks. The lack of significant differences in major life events and the absence of pre-existing panic attacks support the hypothesis that the IVA scenario had a direct psychological impact on the responders. 

These findings are consistent with prior research, such as the study on first responders to the 2004 Madrid terrorist attack, which reported a heightened prevalence of panic attacks (13.9%) within 5–15 weeks of the incident [39]. The significance of these findings lies not only in the elevated rate of panic attacks but also in their potential association with other psychological disorders [40]. Despite this, research specifically addressing panic attacks among emergency service personnel (ESP) remains limited. This gap underscores the need for targeted, panic-specific interventions to address the identified trends [24]. Moreover, the research found no significant differences in the prevalence of GAD or PTSD (0%) among firefighters. Additionally, the PTSD subscales showed no significant differences between the groups. The perceived trends observed in the Arousal subscores (*p* = 0.041) and Total PCL-5 scores (*p* = 0.043) suggest higher scores in the comparison group (CG) relative to the experimental group (DG). While these results align with the suggestion that the control group appears to be at a higher risk of PTSD symptoms, we would like to clarify that the differences observed did not reach statistical significance after applying Bonferroni adjustment (significance level: *p* = 0.01). This adjustment was made to account for multiple comparisons and reduce the likelihood of Type I errors, ensuring the robustness of our findings.

The absence of PTSD cases among these firefighters is at first somewhat surprising given the documented stressors related to such incidents [9,29].

One possible explanation is that the responders may have perceived the incident as a high-stress traffic accident, with the IVA already being over by the time they arrived. It was, rather, an exposure to secondary traumatization, as they were not directly involved in the IVA. Debriefings led by the emergency aftercare team (ENT) and subsequent follow-ups provided a controlled environment for responders to process their experiences shortly after the event [19]. Another potentially positive factor is the digital platform “Charly BOS”, a training system designed to prevent trauma sequelae among civilian emergency responders, which has already been implemented in the Berlin Fire Department. This system has been shown to improve self-assessment regarding PTSD in civilian responders, such as firefighters and police officers [20]. This approach enhances awareness and adaptive responses to stress among firefighters, which serves as a key resilience factor [41,42]. This proactive approach likely helped in reducing the risk of chronic trauma symptoms like PTSD. Indeed, trauma exposure does not always directly result in PTSD [43]. For instance, the 2004 Madrid [39] study reported a low PTSD prevalence (1.2%) among ESP, suggesting that while PTSD may be averted, panic attacks as a prolonged emotional response and other psychological symptoms are sometimes overlooked in traditional symptom assessments [2,28,44].

We aimed to explore the concept of hostility, building on the work of Wesemann et al., who investigated hostility among police officers following the 2016 Christmas market terrorist attack in Berlin [45]. Their findings suggested that police officers exhibited heightened levels of hostility after the event, potentially due to the differences in tasks between emergency responders, with police officers focusing on the perpetrators rather than victims.

In this context, we sought to examine whether assuming non-occupational tasks, such as police duties or responsibilities outside their specific training, could also lead to increased hostility in firefighters. Our data revealed that firefighters who responded to the IVA and took on non-occupational tasks showed a correlation with increased hostility.

These tasks, reported in our sample, included activities such as police investigations, patient assessments (triage), and crime-scene cleanup.

Several factors may have contributed to this heightened hostility. Role conflicts and role ambiguity were likely significant factors [46]. When firefighters were assigned tasks outside their usual responsibilities, such as engaging in police investigations or triage, confusion was created regarding their roles. The lack of clarity about their duties, combined with high-stress circumstances, could have led to a sense of being overwhelmed, fostering hostility toward the situation and those assigning the tasks [47,48]. Additionally, feelings of incompetence may have been a contributing factor. Firefighters, who are trained for specific emergency response duties, may have felt insecure or inadequate when tasked with roles outside their training, such as conducting assessments of victims (triage) or managing law enforcement responsibilities. The perception of intrusiveness also seems relevant in this context. Firefighters may have felt that being asked to take on responsibilities traditionally handled by police or medical professionals was an overreach. This could have led to a sense of being “pushed” into roles and feeling responsible for tasks that exceeded their professional competence, which could have led to frustration, unfairness and the sense that they were abandoned by the organization, contributing to hostility towards the organization and higher authorities.

Overwhelming stress, which can lead to aggression, emotional exhaustion, or even cynicism—all linked to hostility [49]—seems to have contributed to the increased hostility observed among firefighters [50]. Although intervention approaches already exist in the research [51], the area of secondary traumatization among ESP remains an underexplored topic. Therefore, it is important to monitor this issue closely, and further research should explore whether non-occupational tasks lead to similar outcomes in other critical incidents. This is particularly relevant, as such issues could be addressed organizationally by raising awareness among incident commanders to ensure that their teams are only tasked with duties they are specifically trained for. Alternatively, providing training for more specialized tasks, such as patient assessments or psychological preparation before being directly involved in crime-scene cleanup, could help mitigate further psychological issues [21,22,23].

We hypothesized that firefighters who are parents might experience heightened stress during incidents involving child victims given the potential for emotional identification with the victims and their families, as well as the general consensus among firefighters that such incidents are among the most distressing [9]. We investigated GAD and panic attacks due to their strong correlation with PTSD and the limited attention they have received in the literature compared to PTSD [24,25,26]. However, our results showed no significant difference in the prevalence of panic attacks or generalized anxiety between firefighters with and without children. This finding suggests that the presence of children among the victims may not necessarily have exacerbated psychological stress for firefighters who are parents compared to those who are not. However, given the very limited research on this topic, further investigation is essential to determine whether emergency service personnel with children experience heightened stress during incidents involving children. Furthermore, screening for mental health issues should be implemented as early as the start of firefighter training, similar to the approach used for US soldiers. However, conducting a prospective study on this topic presents its challenges [52].

Finally, while our study highlights the psychological burden faced by firefighters in relation to secondary traumatization from the IVA scenario, it is important to note that stress factors are often similar across various emergencies, including terrorist attacks, natural disasters, and large-scale accidents. Research conducted during the COVID-19 pandemic, for example, has demonstrated the significant challenges and negative impact on the mental health of ESP during this time [16,17]. Therefore, it is crucial to examine the effects of diverse emergencies on the mental health of responders, shedding light on the wide-ranging challenges they face.

## 5. Limitations

In this research approach, we used self-report questionnaires. To enhance the depth of insights, future investigations should consider utilizing clinical interviews to validate the diagnosis of mental disorders among firefighters. While resilience capabilities are crucial in mitigating the effects of stress, they were not investigated in this study. Similarly, hardiness—negatively associated with exhaustion and cynicism—represents a promising intervention approach. Training coping strategies that emphasize personal control and competence could help firefighters manage high-stress situations more effectively, enabling them to regain a sense of control over their responses [49,53]. We acknowledge these points as limitations, as our focus was primarily on the psychological symptoms of emergency workers following secondary traumatization.

Another limitation is the small sample size, which resulted from the unique nature of the single event investigated. Therefore, analyzing differences in psychological symptoms between subgroups, such as firefighters with and without children (parenthood), risks being statistically underpowered.

Although we asked firefighters about other critical life events in the past five years, the absence of baseline data—specifically, an assessment of their mental health prior to the event—and the period between the critical incident and the survey are significant limitations. Due to this, the study design relied on a post-test comparison group, which is methodologically weak. Collecting baseline data at the start of firefighters’ training would have provided a clearer reference point and improved comparability between groups. Future studies should include regular monitoring of psychological health to offer a more comprehensive understanding of the impact of traumatic experiences.

Additionally, a longitudinal approach examining time points 12 and 24 months after the critical event is planned for future research to offer a more comprehensive understanding of the long-term effects over time.

## 6. Conclusions

Our study found that firefighters on duty at the IVA in Berlin in 2022 experience a significantly higher rate of panic attacks, indicative of secondary traumatization, while the prevalence of PTSD remains at 0%. These findings highlight the need to broaden mental health support frameworks to address not only PTSD but also other psychological symptoms, such as panic attacks. Identifying exposure-specific factors can help us better understand which firefighters may be at higher risk, allowing us to target preventive measures more effectively. Additionally, non-occupational tasks assigned to firefighters during emergencies were correlated with increased hostility, suggesting the importance of clear role definitions and appropriate task distribution to mitigate psychological distress.

Our findings also emphasize the value of resilience training and proactive mental health support, particularly interventions focusing on coping strategies that promote personal control. While the presence of children among victims did not seem to exacerbate stress for firefighter parents, further research on this topic is needed. Additionally, regular psychological health monitoring should be integrated into firefighter training programs to better assess and address the long-term effects of trauma exposure. This will help ensure that mental health support remains effective, targeted, and responsive to the evolving challenges faced by emergency service personnel.

## Figures and Tables

**Table 1:** *t*-test for independent samples to compare the deployed group and the comparison group with respect to age and years of service (YoS).

	Group	*n*	Mean	Standard Deviation	*t*-Value	*p*-Value
Age	DG	58	39.03	10.55	0.80	0.425
	CG	52	37.33	11.81		
YoS	DG	59	11.39	8.32	0.46	0.649
	CG	53	12.30	12.23		

Notes. YoS = years of service, DG = deployed group, CG = comparison group.

**Table 2:** *t*-test for independent samples comparing PCL-5 subscale scores between the deployed group and the comparison group.

Subscores	Group	*n*	Mean	Standard Deviation	*t*-Value	df	*p*-Value
PCL Intrusions	DG	60	0.9500	1.29438	−1.323	111	0.188
	CG	53	1.3396	1.81811	-	-	-
PCL Avoidance	DG	60	0.3500	0.63313	−1.417	112	0.159
	CG	54	0.5556	0.90422	-	-	-
PCL Negative Cognitions	DG	60	1.0667	1.48286	−1.280	112	0.203
	CG	54	1.4630	1.81932	-	-	-
PCL Arousal	DG	60	1.7000	2.28703	−2.063	112	0.041
	CG	54	2.6852	2.80716	-	-	-
PCL Total Score	DG	60	4.0667	4.59870	−2.047	112	0.043
	CG	54	6.0185	5.57112	-	-	-

Notes. DG = deployed group, CG = comparison group, df = degrees of freedom. Significance level: *p* = 0.01 (Bonferroni-adjusted).

## Data Availability

The data supporting the reported results are not publicly available due to privacy and ethical restrictions. However, they are available from the corresponding author upon reasonable request and with permission of the Bundeswehr Hospital Berlin.
